# Mitochondrial Heterogeneity: Evaluating Mitochondrial Subpopulation Dynamics in Stem Cells

**DOI:** 10.1155/2017/7068567

**Published:** 2017-07-05

**Authors:** D. C. Woods

**Affiliations:** Laboratory of Aging and Infertility Research, Department of Biology, Northeastern University, Boston, MA 02115, USA

## Abstract

Although traditionally viewed as the “powerhouse” of the cell, an accruing body of evidence in the rapidly growing field of mitochondrial biology supports additional roles of mitochondria as key participants in a multitude of cellular functions. While it has been well established that mitochondria in different tissues have distinctive ultrastructural features consistent with differential bioenergetic demands, recent and emerging technical advances in flow cytometry, imaging, and “-omics”-based bioinformatics have only just begun to explore the complex and divergent properties of mitochondria within tissues and cell types. Moreover, contemporary studies evaluating the role of mitochondria in pluripotent stem cells, cellular reprogramming, and differentiation point to a potential importance of mitochondrial subpopulations and heterogeneity in the field of stem cell biology. This review assesses the current literature regarding mitochondrial subpopulations within cell and tissue types and evaluates the current understanding of how mitochondrial diversity and heterogeneity might impact cell fate specification in pluripotent stem cells.

## 1. Mitochondrial Structure and Diversity

Mitochondria are ubiquitous across eukaryotic organisms and are critical for cellular bioenergetics. The classical mitochondrial ultrastructural features, consisting of an outer membrane and an inner membrane containing invaginations that comprise the matrix-rich cristae, are specifically modified in a tissue-specific manner in order to meet cellular-explicit energy demands. The differences in mitochondrial morphology between cell and tissue types as well as in localization and distribution within the cell have been well documented [1, 2], and more recently, it has been demonstrated that even within a single cell, mitochondrial features fluctuate rapidly in response to alterations in a metabolic state [3, 4]. Although the exact molecular mechanisms underlying ultrastructural remodeling of mitochondria are a topic of investigation with much remaining to be discovered, the diversity observed in a mitochondrial phenotype includes a range in size (from 0.1 micron to 1.0 microns in diameter), shape (from spherical to elongated and tubular), and cristae density (from essentially devoid of cristae to dense cristae). As a mitochondrial form relates to function, early studies postulated that mitochondrial cristae density serves to increase the surface area, thereby enhancing oxidative phosphorylation [5]. With subsequent efforts identifying the inner membrane as containing the components of the electron transport chain and the capacity for ATP generation positively correlated with the cristae surface area (reviewed in [6]), the relationship between energy production and mitochondrial morphology, with specific modifications in the inner membrane and cristae structure, has become increasingly clear. For example, ATP synthase dimerization has been demonstrated to govern biogenesis of the inner membrane and cristae formation, and oligomerization of F1FO-ATP synthase has been proposed to mediate the formation of cristae by modulating inner membrane curvature [7–10]. Additionally, a number of candidate proteins involved in modulation of cristae formation and inner membrane organization have been identified across model organisms and specifically correlated with pathophysiological disease states in humans (reviewed in [5]). Mitochondrial morphological dynamics are also dependent upon fission and fusion of inner and outer membranes, mediated by large GTPases [11–13], including the cytosolic DRP1 [14], the mitofusin (MFN) proteins 1 and 2 (MFN1 and MFN2) [15], and optic atrophy 1 (OPA1) [16–19].

**Figure 1:**
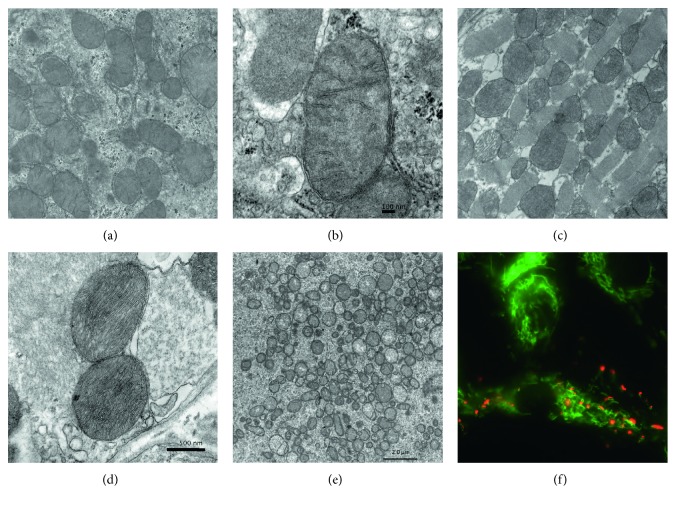
Mitochondrial heterogeneity between tissue types and within cells. (a)–(e) Transmission electron micrographs depicting mitochondrial features in mouse liver ((a), enlarged in (b)), mouse heart ((c), enlarged in (d)), and a primary-stage human oocyte (e). (f) A fluorescent micrograph depicting JC-1-labeled KGN cells demonstrates heterogeneity in mitochondrial membrane potential between cells and between individual subcellular mitochondrial populations. JC-1 monomers (green) indicate low Δ*ψ*m and JC-1 aggregates (red) indicate high Δ*ψ*m. Scale bars as marked.

Although the specifics regarding how these nuclear-encoded proteins have been shown to participate in mitochondrial remodeling have been studied for almost two decades and extensively reviewed elsewhere [4, 20], far less is known regarding the cell signaling mechanisms that govern a mitochondrial phenotype and how this occurs on a tissue-specific basis during the process of differentiation. In undifferentiated embryonic stem cells (ESCs), mitochondria are characterized as having a globular shape, with few defined cristae and limited oxidative capacity [21]. This ovoid mitochondrial morphology and cristae arrangement are rather striking [21, 22] and also observed in oocytes (Figure 1), as well as in the inner cell mass of blastocysts—the originating source for ESCs [23]. The low matrix and cristae density are associated with slowly respiring state IV mitochondria, as compared to fast respiring state III mitochondria, which have a higher matrix and cristae density and a morphologically “condensed” ultrastructure [24–26]. Notably, the low cristae density and ovoid structure, along with distinctive perinuclear localization, are under consideration as features for cellular “stemness,” having been detected in adult somatic stem cells and reprogrammed stem cells (i.e., iPSCs [21, 27, 28]), in addition to numerous ESC lines. Intriguingly, observations of the modification of mitochondrial patterning in a spontaneously differentiating rhesus monkey adult mesenchymal cell (MSC) line (ATSC line) have demonstrated heterogeneity within stem cell populations and have led to the postulation that mitochondrial localization (deviation from a perinuclear region) may be a mechanism by which to monitor differentiation status, in vitro [28], although this remains to be firmly established, and applicability may be cell type- and status-dependent, as perinuclear localization can be induced in disease states and under conditions of hypoxia and apoptosis in nonstem cell lines [29–31]. A detailed analysis of several nonstem cell types, including HeLa, HUVECs, COS-7, cortical astrocytes, and primary hepatocytes, also demonstrated heterogeneity with respect to both distribution and functional properties [32]. Although in each cell type examined, mitochondria were distributed throughout the cell body, both cortical astrocytes and HUVECs demonstrated distinct perinuclear clustering, which was less pronounced in HeLa and COS-7 and absent in primary hepatocytes [32]. Functionally, it was noted that each cell type exhibited heterogeneity in mitochondrial membrane potential (potential (Δ*ψ*m)), as each cell type examined contained distinct populations of mitochondria having both high or low Δ*ψ*m, and no single mitochondrion contained regions of high and low Δ*ψ*m. Quantitative analysis of Δ*ψ*m in HeLa cells demonstrated that mitochondria localized to the perinuclear region had a greater percentage of mitochondria with low Δ*ψ*m and were more closely associated with endoplasmic reticulum (ER) than their peripherally located counterparts [32].

Morphological changes in the physical appearance of mitochondria during the process of differentiation are not well characterized, although it is well understood that mitochondrial ultrastructure varies dramatically between tissue types ([1], Figure 1). In a recent study evaluating the changes in mitochondrial features that accompany progressive states of MSC differentiation into the endothelial lineage, Shin et al. assessed mitochondrial numbers, length, resident area per cell, and morphology. Consistent with the high energy demands required for differentiation followed by a subsequent decline as cells approach a terminally differentiated phenotype, the authors determined that mitochondrial number, area per cell, length, and morphological complexity decreased corresponding with progression of differentiation [33]. To specifically address the mitochondrial reconfiguration that occurs during the differentiation process, Forni et al. monitored changes in mitochondrial mass, morphology, dynamics, and bioenergetics during MSC differentiation into osteocyte, chondrocyte, and adipocyte lineages. Data indicate that mitochondrial elongation and increases in MFN1 and MFN2 occurred during the early stages of adipocyte and osteogenic differentiation, whereas chondrogenesis was associated with a fragmented mitochondrial phenotype. Strikingly, the differentiation ability of MSCs was inhibited following knockdown of Mfn2 in adipogenesis and osteogenesis, while dominant-negative Drp1 impeded the chondrogenesis differentiation capability [34]. Together, these data provide strong supporting evidence for a fundamental role for mitochondrial dynamics, including the modulation of mitochondrial ultrastructure, in the differentiation process.

Although metabolic and structural remodeling during cell differentiation represents emerging fields of study in mitochondrial and stem cell biology, mitochondrial heterogeneity as it pertains to specific mitochondrial subpopulations within a single tissue or cell remains a considerably under-characterized component in cell biology. It is well understood that under physiological conditions, cells possess a heterogeneous mitochondrial population based on differences in membrane potential (Δ*ψ*m [32, 35, 36]). Mitochondria with a low Δ*ψ*m are generally regarded as metabolically quiescent (“resting” or damaged), whereas those with a high Δ*ψ*m are viewed as metabolically active (“respiring”). However, there are caveats to this generalization, including that Δ*ψ*m is maintained by the balance between the electron transport chain ATP synthases. Accordingly, even well-coupled (respiring) mitochondria can have a decreased Δ*ψ*m. The heterogeneity in Δ*ψ*m both within individual cells and between neighboring cells can be observed in vitro using fluorometric compounds, such as JC-1 (5,5′,6,6′-tetrachloro-1,1′,3,3′-tetraethylbenzimidazolylcarbocyanine iodide). JC-1 undergoes a shift in spectral fluorescence from green (in mitochondria that are metabolically inactive) to red-orange upon conversion from monomeric (green) to aggregate (red-orange) form in actively respiring mitochondria. Utilizing the aggregate fluorescent properties of JC-1 to detect differences in mitochondrial respiration status in undifferentiated human ESCs (H9), Kumagai et al. have recently demonstrated that shifts in Δ*ψ*m (e.g., cells with more aggregate-state JC-1) within undifferentiated ESC colonies may serve as a simple visual predictive indicator of cells that are undergoing the earliest stages of differentiation [37]. Due to the predominant metabolic reliance on glycolysis during the undifferentiated state [38–41], the observed increase in Δ*ψ*m associated with oxidative phosphorylation is one of the first markers to distinguish differentiating cells in a mixed culture. Accordingly, mitochondrial metabolic changes have been proposed as early markers of stem cell differentiation [38–41], although how these changes occur on a cell type-specific basis and the relationship of metabolic shifts to cell fate specification remains less well defined. However, it has been shown that mitochondrial activity and stem cell function are intimately linked. In isolating undifferentiated mouse ESCs based on differences in Δ*ψ*m, Schieke et al. obtained two distinct populations of ESCs which were indistinguishable based on morphology, yet had remarkably different resting oxygen consumption rates (low Δ*ψ*m, Δ*ψ*mL; high Δ*ψ*m, Δ*ψ*mH). Analysis of differentiation potential revealed that in the presence of bone morphogenetic protein 4 (BMP-4) in the absence of leukemia inhibitory factor (LIF) to promote mesoderm specification, Δ*ψ*mL ESCs exhibited a markedly greater mesodermal differentiation capacity (10-fold) than Δ*ψ*mH ESCs, whereas Δ*ψ*mH ESCs had a greater propensity for teratoma formation than Δ*ψ*mL ESCs [42]. Furthermore, treatment with rapamycin, a potent inhibitor of mTOR, resulted in a decrease in Δ*ψ*m and oxygen consumption in undifferentiated mouse ESCs and augmented BMP-4-induced mesodermal differentiation [42]. Together, these data suggest a strong link between intrinsic mitochondrial metabolic function and stem cell fate.

Alterations in Δ*ψ*m in human ESCs have also been demonstrated as a consequence of in vitro age (e.g., passage number). In undifferentiated H9 and PKU1 cell lines, Δ*ψ*m increased significantly in late-passage cells as compared to their younger counterparts. The elevated Δ*ψ*m also correlated with increases in total mitochondrial volume and generation of reactive oxygen species (ROS) [43]. Late-passage human ESCs also exhibited a reduced capacity for differentiation. While early-passage cells differentiated evenly into ectodermal, mesodermal, and endodermal lineages, high-passage cells preferentially differentiated into ectodermal lineage, although the authors acknowledge that the impact of prolonged duration on culture cannot be ruled out, rather than directly related to the passage number of the cell line [43]. Given that additional established markers of stemness, such as telomerase activity and pluripotency markers, were unaffected by passage number, the impact of in vitro age on mitochondrial function is striking and may represent an additional factor when screening stem cells for potential therapeutics. This type of in vitro aging has a similar impact on the mitochondria of iPSCs. In a direct comparison between iPSCs cultured for 1 month postcellular reprogramming (young iPSCs) and iPSCs cultured for over 1 year (aged iPSCs), H_2_O_2_-dependent Δ*ψ*m depolarization occurred at a faster rate in aged iPSCs than that observed in their “younger” counterparts, demonstrating a diminished ability to counteract oxidant exposure with in vitro age. Moreover, the capacity for in vitro neurogenesis was diminished in the aged iPSCs versus the young iPSCs [44].

Although emerging evidence supports a role for Δ*ψ*m in the maintenance of stemness, the impact of mitochondrial subcellular heterogeneity on cellular function, including differentiation, has not been evaluated. Based on a series of studies examining mitochondrial properties within individual cells [34, 35], Kuznetsov and Margreiter have neatly described mitochondrial heterogeneity as belonging to 4 major classifications (reviewed in [45]): (1) *ultrastructure*—mitochondria range in size (0.2 *μ*m to 1.0 *μ*m), shape (circular to elongated and tubular), and cristae density (no visible cristae/vacuole-like appearance to dense cristae) and can be found alone or physically networked; ultrastructure varies across tissue types, as well as within individual cells; and fission-fusion events, as well as respiratory status, also impact on morphology; (2) *functional properties*—mitochondria can differ in redox state, respiration, intramitochondrial Ca^2+^ and reactive oxygen species (ROS) levels, mitochondrial protein composition and content, and Δ*ψ*m; (3) *behavior*—mitochondria respond differently to oxidative stress, starvation, apoptotic stimuli, and mitophagy signals and exhibit selective responses to toxins and substrates; (4) *dynamics*—cell type-specific intracellular localization, oscillatory movements, translocation events, filament extension and retraction, and fission and fusion events [45]. Additionally, new evidence also now demonstrates heteroplasmy in mitochondrial DNA (mtDNA) at the level of the single cell [46], bringing another layer into subcellular mitochondrial heterogeneity. However, despite the understanding that mitochondria within a cell may well serve different functions, precise characterization of subcellular mitochondrial subpopulations has proven challenging, due to difficulties in the isolation of specific mitochondrial subtypes for further analysis [45–47], and much remains to be discovered. In a discussion of mitochondrial heterogeneity pertaining to Δ*ψ*m, Wikstrom et al. propose that low Δ*ψ*m represents a mechanism by which mitochondria are selectively targeted for autophagy [36]. Although the dynamics that define mitophagy are unclear, it has been demonstrated that mitochondria depolarize prior to autophagy [48, 49] and join a preautophagic mitochondrial pool [49] characterized by small size and reduced levels of the mitochondrial fusion protein, OPA1 [49, 50]. It has also been proposed that mitochondrial heterogeneity may contribute to preservation, as reduced metabolic activity may serve to preserve genomic integrity [42].

## 2. Tissue- and Cell-Specific Mitochondrial Subpopulations

In addition to the differences in mitochondrial morphology mentioned above, mitochondria also differ functionally and are known to be involved in cellular processes beyond metabolism. Such functions are well characterized and ubiquitous, including cell death and differentiation, intracellular Ca^2+^ regulation, oxygen sensing, and ROS generation, while others are cell and tissue type-specific, such as steroid hormone biosynthesis, hormone signaling and responsiveness, thermogenesis, hemesynthesis, and processing of toxins [51]. These complex processes, in addition to those associated with metabolism, are carried out through bidirectional communication with the nucleus. In humans, 13 proteins, along with 2 rRNAs and 22 tRNAs, are encoded by the 37 genes contained within the small (16,569 bp) circular mitochondrial genome. The proteins serve as key constituents of mitochondrial electron transport chain (ETC) protein complexes I–IV that are embedded in the inner mitochondrial membrane. However, mitochondrial protein composition is estimated to approach 1500 proteins [52] encoded by the nuclear genome, presumably to execute the diverse array of functions performed by mitochondria. Of these, approximately 1000 proteins have been identified (although not functionally characterized) [53], with the vast majority predicted to localize to the mitochondrial inner membrane and the matrix [54, 55]. However, mitochondrial protein composition is not fixed, nor is it consistent between tissue types. A well-executed proteomic study using ultrapurified mitochondrial preparations revealed striking compositional differences between brain, kidney, liver, and heart mitochondria, determining that only 57% of mitochondrial proteins identified were consistently expressed between the examined tissues [55]. With nearly half of the protein composition of mitochondria differing between tissue types, the cell type-specific differences in mitochondrial function are also underscored. Although mitochondria are highly morphologically heterogeneous between tissues [1, 56], details regarding proteomic profiles on mitochondrial subpopulations within a single cell or tissue type are lacking, as is information regarding the ESC mitochondrial proteome [57]. Mitochondrial proteomic analyses in pluripotent stem cells as they undergo transformational changes related to reprogramming and differentiation would likely provide important information pertaining to the utility of stem cells in research and, as directed differentiation strategies improve, therapeutics and regenerative medicine.

Given the complexity and diversity of mitochondrial proteomic profiles amongst mitochondrial subtypes, it is not surprising that mitochondria serve to perform a diverse spectrum of functions—with many more remaining to be discovered. Among the more well-characterized mitochondrial types within a single tissue are two cardiac mitochondrial subpopulations, subsarcolemmal mitochondria (SSM) and interfibrillar mitochondria (IFM). Initially characterized by ultrastructural differences observed between the two populations located beneath the sarcolemma and between the myofibrils, respectively, physical isolation of each mitochondrial subtype revealed distinct biochemical functional properties [58]. Specifically, succinate dehydrogenase and citrate synthase activities were elevated in the IFM population as compared to SSM, and oxidation of substrates was found to proceed 1.5× faster in IFM isolates than SSM preparations [58]. Subsequently, the Ca^2+^ uptake capability of IFM and SSM was comparatively demonstrated to differ, as a further indicator of mitochondrial heterogeneity between the two subpopulations [59], although differences in isolation strategies may have impacted some of these conclusions. Additionally, IFM demonstrated higher protein import rates for the precursors to malate dehydrogenase and ornithine carbamoyltransferase [60]. However, due to the technically difficult nature of the isolation procedures, in-depth comparative experimental characterization between the two subtypes has been impeded. More recently, Hatano et al. have developed a three-dimensional computational model, integrating electrophysiology, metabolism, and mechanics with subcellular structure. Using this intriguing multifaceted simulation approach, the authors demonstrate that the impact of the subcellular environment modulated mitochondrial function [61]. Although individual intrinsic functional differences between mitochondrial populations could not be examined in the study, this work highlights that mitochondria work within a subcellular “niche,” in which microenvironmental cues can govern function. It is intriguing to think of mitochondrial subpopulations as respondents to the microenvironment (similar to stem cells within a niche). As a first step toward evaluating “mitochondrial heteroplasmy” in a single mitochondrion, Pham et al. developed a microrespirometer to monitor mitochondrial respiration on individual organelles. Using this novel technological approach, the authors confirmed differences in respiration between coupled and uncoupled mitochondria [47]. Additionally, our own data utilizing a nanoparticle-sorting platform for the isolation of mitochondrial subpopulations similarly revealed differences in the ATP-generating capability between coupled and uncoupled mitochondria; however, a subset of uncoupled mitochondria could be induced to generate ATP when the microenvironment was altered [62]. As more information becomes available regarding intrinsic mitochondrial differences, computational models along with emerging technologies to evaluate mitochondrial subpopulations such as this will likely prove invaluable in the development of experimental paradigms and testable hypotheses.

## 3. Conclusion

The current understanding of mitochondrial function in stem cells is limited in scope as compared to the broader field of mitochondrial biology. Given the highly specialized features of mitochondria in differentiated cell types, it stands to reason that a separate field of study dedicated to mitochondrial fate specification and differentiation might coevolve with the stem cell field. In this way, stem cells serve as an excellent model to study “mitochondrial differentiation.” As each mitochondrion contains DNA encoding for only 13 proteins, yet contains a subtype-specific proteomic profile of up to 1500 nuclear-encoded proteins, the significance of nuclear communication in the regulation of mitochondrial function becomes increasingly clear. How the mitochondria and nucleus communicate on a per organelle basis (i.e., why some are activated while others remain resting, how cell type-specific functions are executed) remains to be determined. As nanoscaled technologies emerge for the study of subcellular organelles, the mechanisms that govern mitochondrial heterogeneity and function will be elucidated and perhaps provide additional platforms and metrics for stem cell reprogramming and differentiation.
